# Exploring laccase-like multicopper oxidase genes from the ascomycete *Trichoderma reesei*: a functional, phylogenetic and evolutionary study

**DOI:** 10.1186/1471-2091-11-32

**Published:** 2010-08-24

**Authors:** Anthony Levasseur, Markku Saloheimo, David Navarro, Martina Andberg, Pierre Pontarotti, Kristiina Kruus, Eric Record

**Affiliations:** 1 UMR-1163, INRA de Biotechnologie des Champignons Filamenteux, IFR86-BAIM. Universités de Provence et de la Méditerranée, ESIL, 163 avenue de Luminy, CP 925, 13288 Marseille Cedex 09, France; 2 Universités Aix-Marseille 1 et 2, UMR-1163, 163 avenue de Luminy, CP925, 13288 Marseille Cedex 09, France; 3 VTT Biotechnology: VTT Technical Research Centre of Finland, P.O. Box 1500, Tietotie 2, 02044 VTT Espoo Finland; 4 Evolution Biologique et Modélisation: UMR 6639 CNRS Université de Aix Marseille, 3 place V. Hugo, 13331 Marseille, France

## Abstract

**Background:**

The diversity and function of ligninolytic genes in soil-inhabiting ascomycetes has not yet been elucidated, despite their possible role in plant litter decay processes. Among ascomycetes, *Trichoderma reesei *is a model organism of cellulose and hemicellulose degradation, used for its unique secretion ability especially for cellulase production. *T. reesei *has only been reported as a cellulolytic and hemicellulolytic organism although genome annotation revealed 6 laccase-like multicopper oxidase (LMCO) genes. The purpose of this work was i) to validate the function of a candidate LMCO gene from *T. reesei*, and ii) to reconstruct LMCO phylogeny and perform evolutionary analysis testing for positive selection.

**Results:**

After homologous overproduction of a candidate LMCO gene, extracellular laccase activity was detected when ABTS or SRG were used as substrates, and the recombinant protein was purified to homogeneity followed by biochemical characterization. The recombinant protein, called TrLAC1, has a molecular mass of 104 kDa. Optimal temperature and pH were respectively 40-45°C and 4, by using ABTS as substrate. TrLAC1 showed broad pH stability range of 3 to 7. Temperature stability revealed that TrLAC1 is not a thermostable enzyme, which was also confirmed by unfolding studies monitored by circular dichroism. Evolutionary studies were performed to shed light on the LMCO family, and the phylogenetic tree was reconstructed using maximum-likelihood method. LMCO and classical laccases were clearly divided into two distinct groups. Finally, Darwinian selection was tested, and the results showed that positive selection drove the evolution of sequences leading to well-known laccases involved in ligninolysis. Positively-selected sites were observed that could be used as targets for mutagenesis and functional studies between classical laccases and LMCO from *T. reesei*.

**Conclusions:**

Homologous production and evolutionary studies of the first LMCO from the biomass-degrading fungus *T. reesei *gives new insights into the physicochemical parameters and biodiversity in this family.

## Background

Lignin degradation is a key step for recycling the carbon fixed by photosynthesis, and to date, basidiomycetes are the most efficient naturally-found lignin degraders [1,2]. Lignin attack is a complex oxidative process in which heme peroxidases oxidize lignin subunits using extracellular hydrogen peroxide generated by unrelated oxidases as a co-substrate. A second enzyme group involved in lignin degradation is the multicopper oxidases (laccases) that oxidize lignin subunits with molecular oxygen as the electron acceptor. Different fungal lignocellulose degradation strategies have been reported, and a better understanding of ligninolysis could be achieved by screening the "computational biodiversity" found in fungal genomes. Gene networks could then be highlighted and correlated to the lignin-degrading ability of different fungal strains. For this purpose, dedicated databases, namely CAZy [[3], http://www.cazy.org] and FOLy [[4], http://foly.esil.univ-mrs.fr] were designed to annotate the genes involved in the (hemi)cellulolytic and ligninolytic processes, respectively. In the FOLy database there is an inventory of laccases (benzenediol-oxygen-oxidoreductase, EC1.10.3.2), which are copper-containing oxidases able to oxidize a wide range of aromatic compounds [5,6]. Based on multiple sequence alignments of more than 100 laccases, four ungapped sequences regions (L1-L4) were evidenced in laccases [7]. The copper ligands include 12 amino acids that are housed within these conserved regions. Moreover, four loop regions (I, II, III, and IV) were identified and suggested to be involved in substrate binding on the basis of 3D structure superimposition [8].

Phenols are typical substrates of laccases (syringaldazine, DMP and guaiacol) but laccases are also able to oxidize electron donor substrates such as ABTS [2,2'-azino-bis(3-ethylbenzothiazoline-6-sulfonic acid)] or ferrocyanide [9,10].

Laccases are attractive environmentally-friendly enzymes that have shown potential for a variety of applications. Laccases find potential applications in pulp delignification and biobleaching [11], dye-bleaching in the textile and dye industries [12], treatment of wastewater [13], removal of phenolic compounds in beverages [14], biosensor and biofuel cell construction [15] and products of pharmaceutical importance [16].

Whereas laccases are well-known ligninolytic enzymes in basidiomycetes, their role in ascomycetes is still being unravelled. Potential laccases have been already reported in ascomycetes in which laccases were supposed to be involved in the melanin-like pigment synthesis in conidiospores, in the induction of fruiting bodies, and finally in pathogenic interaction with plants [17-22]. Complete annotations of the potential ligninolytic systems were recently produced using FOLy, providing initial comparative insight into the diversity of fungal lignin degradation [4]. Putative laccase-related genes (called LO1 according our classification) were interestingly identified in *Trichoderma reesei *and other ascomycetes. *T. reesei *is a mesophilic soft-rot ascomycete fungus producing high levels of cellulases and hemicellulases that are also commercially used to modify and hydrolyze plant cell wall polysaccharides. To date, no ligninolytic activity has been reported for this fungus.

This paper reports overexpression of a laccase-like multicopper oxidase gene (LMCO) from *T. reesei *and the biochemical characterization of the recombinant protein. Secondly, phylogenetic reconstruction and evolutionary analyses (testing for positive selection) were performed to explore the biodiversity of this enzyme group in *Ascomycotina*.

## Methods

### Strains

*Escherichia coli *JM 109 (Promega, Charbonnières, France) was used as plasmid host. *T. reesei *strain Rut-C30 [23] was used for homologous overexpression.

### Expression vectors and fungal transformation

After codon optimization, gene|124079| was synthesized, sequence-checked, and ligated in the expression vector pAMH110 after digestion with SacII and NdeI restriction enzymes. In this vector, the *T. reesei *cellobiohydrolase I-encoding gene (*cbhI*) promoter was used to drive the expression of the laccase gene. Fungal transformations were carried out essentially as described previously [24].

### Media and culture conditions

*T. reesei *strains were maintained on potato dextrose agar (Difco, Sparks, MD, USA) slants. Transformants were regenerated on solid minimal medium containing, per liter: (NH_4_)_2_SO_4 _5.0 g, KH_2_PO_4 _15.0 g, CaCl_2 _0.45 g, MgSO_4 _0.6 g, CoCl_2 _3.7 mg, FeSO_4_.H2O 5 mg, ZnSO_4_.H2O 1.4 mg, MnSO_4_.H_2_O 1.6 mg, glucose 20 g as carbon source, sorbitol 182 g as osmotic stabilizer, and hygromycin 125 mg for selection. Plates were solidified and colony growth was restricted by adding 2% agar to the medium. Transformed protoplasts were plated in 3% selective top agar containing 1 M sorbitol.

For screening of laccase activity on plates, transformants were plated on solid minimal medium with 200 μM ABTS. The plates were incubated for 7 days at 30°C and checked for development of a purple halo. In the shake flask cultivation, the transformants were grown in minimal medium containing, per liter: (NH_4_)_2_SO4 5.0 g, KH_2_PO_4 _15.0 g, CaCl_2 _0.6 g, MgSO_4 _0.6 g, CoCl_2 _3.7 mg, FeSO_4_.H_2_O 5 mg, ZnSO4. H_2_O 1.4 mg, MnSO_4_.H_2_O 1.6 mg, peptone 5 g, lactose 40 g, Solka floc cellulose (International Fiber Corporation, North Tonawanda, NY, USA) 20 g as carbon sources and inducers, and piperazine-N,N'-bis(2-ethanesulphonate) 33 g. The pH was adjusted to 5 with KOH. The culture medium was inoculated with 1 × 10^7 ^spores per 50 ml and grown in baffled flasks at 30°C with stirring at 200 rpm. Aliquots (1 mL) were collected daily from liquid culture medium and cells were removed by filtration (0.45 μm). Laccase activity was subsequently measured.

### Laccase activity measurement

In the culture medium, laccase activities were determined quantitatively by monitoring the oxidation of 500 μM ABTS at 420 nm (extinction coefficient, 36000 M^-1^cm^-1 ^) in the presence of 50 mM Na-K-tartrate, pH 4.0 at 30°C. For optimal pH determination, laccase activity was also measured by following the oxidation of syringaldazine (SRG) [N,N'-bis-(3,5-dimethoxy-4-hydroxybenzylidene)hydrazine] to quinone (ε = 65 000 M^-1^·cm^-1^) at 525 nm [25].

### Purification and protein characterization

The best-producer transformant was inoculated in the same conditions as in the screening procedure. Culture was harvested after 7 days of growth, filtered (0.7 μm), and concentrated by ultrafiltration through a polyethersulfone membrane (molecular mass cut-off: 30 kDa) (Millipore). The concentrate was dialyzed against the binding buffer (30 mM Tris-HCl, pH 7.0) and His-tagged recombinant protein was purified on a Chelating Sepharose Fast Flow column (13 × 15 cm; Amersham Biosciences) previously charged with 0.4 M NiSO_4 _solution, and equilibrated with five column volumes of binding buffer. After extensive wash with binding buffer, bound proteins were then eluted with 3 column volumes of an imidazole gradient (0-150 mM) in binding buffer at a flow rate of 1 mL.min^-1 ^and collected with fractions of 5 mL.

### Protein analysis

Protein concentration was determined according to [26] using bovine serum albumin as standard. Protein purification was followed by SDS/PAGE on 11% polyacrylamide slab gels that were then stained with Coomassie blue.

### Temperature and pH optimum

Laccase activity of the purified TrLAC1 was assayed at various setpoint temperatures (range: 30°C to 60°C). For pH, laccase activity was assayed in 50 mM citrate/100 mM phosphate buffer (pH 2.5-7.0), in 50 mM phosphate buffer (pH 6-8) and in 50 mM Tris buffer (pH 9) at 30°C. ABTS was used as substrate in both experiments, and syringaldazine was used to determine optimal pH.

### Temperature and pH stabilities

The effect of temperature on enzyme stability was studied by incubating pure enzyme from 30 min to 24 h at temperature ranging from 30°C to 50°C. After this treatment, residual enzyme activity was determined under standard conditions. The effect of pH on enzyme stability was studied by incubating pure enzyme for 30 min to 48 h at pH ranging from 3.0 to 7.0 in 50 mM citrate/100 mM phosphate buffer (pH 2.5-7.0) and in 50 mM phosphate buffer (pH 6-8) at 4°C. After this treatment, residual enzyme activity was determined under standard conditions.

### Circular dichroism spectroscopy

Circular dichroism (CD) spectra were recorded on a JASCO model J-720 CD spectrometer equipped with a PTC-38WI Peltier thermally controlled cuvette holder. Far-UV (240-190 nm) CD measurements were performed with 2 μM enzyme in 10 mM sodium phosphate buffer pH 7.1 at 25°C, using a 1-mm cell and a bandwith of 1 nm. Spectra were accumulated four times and the values were corrected for buffer contributions.

### Construction of the phylogenetic tree

From the query sequence (gene|124079| reference to *T. reesei *genome database, JGI: http://genome.jgi-psf.org/Trire2/Trire2.home.html), a dataset of putative homologous sequences was built by BLAST [27] run on the Non Redundant database. The raw dataset was manually filtered to eliminate potentially non-homologous sequences, disturbing alignments and duplicates. Sequences retrieved were only focused on scope TaxeID = 4751 (fungal kingdom). An alignment was created using MUSCLE [28] and large gaps were manually eliminated. A default parameter for gap columns thresholds was set at 0.85. A bias correction phase was used to eliminate: i) sequences with a diverging composition, using an amino-acid composition test bundled with TREE-PUZZLE software [29] with an alpha risk set to 5%. From this alignment, a phylogenetic tree was generated using maximum likelihood methods [30]. Bootstrapping was carried out with 1000 replications.

### Evolutionary analyses: testing for positive selection

Protein and DNA sequences were retrieved from the National Center for Biotechnology Information. Protein sequences were aligned using MUSCLE [28]. Correspondence between protein alignment and each DNA sequence was established using Wise2 software followed by manual adjustments [31]. The final alignment contained 290 codons for the dataset.

The codeml program of the PAML (Phylogenetic Analysis by Maximum Likelihood; [37];) 3.15 software package was applied to test for positive selection. PAML uses a maximum likelihood algorithm to assign likelihood scores to different models for selection. If a model incorporating positive selection gave a higher likelihood score than a null model without positive selection, this constitutes evidence for positive selection. Model A implemented by Yang & Nielsen was used [33]. This model enables ω (= dN/dS) to vary both between sites and between lineages, and was implemented in the maximum likelihood framework. Branch A tested for positive selection was labelled as foreground branch, and all remaining branches were labelled as background branches. This model was then used to construct two likelihood ratio tests (LRTs) by comparison against models that do not identify positive selection. The null hypothesis for test 1 is the site model M1a [34,35] which assumes two site classes with 0 < ω0 < 1 and ω1 = 1 for all branches. For test 2, the null hypothesis is the branch-site model A but with ω2 = 1 fixed. Positively-selected sites were identified by the Bayes empirical Bayes (BEB) method [34].

## Results

### Genome annotation of the FOLymes in *T. reesei *QM6a

Genome annotation of *T. reesei *QM6a was performed using the FOLy database. Annotation was focused on enzymes potentially involved in the degradation of lignin and related aromatic compounds. Surprisingly, 6 genes were annotated as laccase-like multicopper oxidase encoding genes (LMCO): jgi|124079|, jgi|122948|, jgi|5119|, jgi|54239|, jgi|102820| and jgi|121098|. Among these candidate genes, model 124079 was selected for further functional investigation for its characteristic motif identification and sequence similarity with classical laccase proteins. This gene is 2193 bp in length and contains 5 introns. The genomic context of this gene was studied to exclude the possibility that it is a pseudogene. The promoter sequence contained CAAT and TATAA boxes at -658 and -109, respectively. Searches for specific regulatory elements revealed 21 heat-shock elements (HSE) and one nitrogen metabolite regulation (NIT2-like). Interestingly, two putative metal response elements and one xenobiotic response element involved in laccase gene regulation in white-rot fungi were identified but located downstream of the ATG (+41; +102 and +11, respectively). The functionality of these regulatory elements needs to be experimentally verified. The corresponding protein, called TrLAC1, contains 623 aa and possesses a classical signal peptide of 20 aa. Structure prediction of TrLAC1 is similar to laccases from basidiomycetes such as *Trametes *species and *Pycnoporus cinnabarinus *(data not shown).

### Homologous overexpression of the laccase-like gene|124079|

Codons of gene|124079| were optimized to perfectly fit the general *Trichoderma reesei *codon usage. Non-optimized and optimized DNA sequences showed an identity of 77,9%. The signal sequence of the laccase genes itself was used in the expression cassette. Six histidine codons were introduced to the C-terminus to provide a tag for subsequent protein purification. Gene expression was regulated by the inducible *cbh1 *promoter and *chb1 *terminator. *T. reesei *RUTC-30 was co-transformed with the genetic cassette including gene|124079|cloned in the expression vector PAMH110, and a vector containing the hygromycin resistance selection marker. Transformants were selected for their ability to grow on solid minimum medium containing hygromycin. Transformants were first screened on plates based on the appearance of purple halos flagging ABTS oxidation. Secondly, approximately 125 colonies were screened by assaying laccase activity in the culture medium, and the best producer was selected for further analysis. The best-producer transformant was then cultured to study the time course of laccase activity. Laccase activity against ABTS was detected on day 2 and the activity increased until day 6 having the maximum activity of 0.6 nkat.mL^-1^. According to the specific activity determined for the purified enzyme, production yield was 280 mg.L^-1^.

### Characterization of the recombinant TrLAC1 protein

#### Molecular mass and protein identification

**Figure 1:**
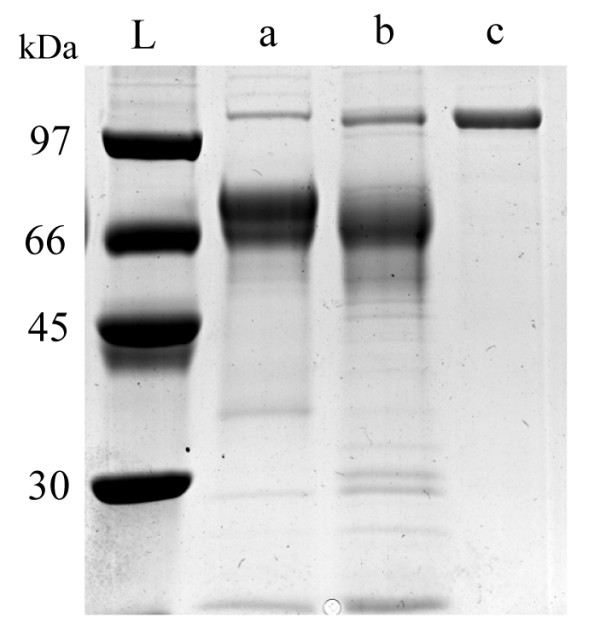
**SDS-PAGE of extracellular proteins and purified TrLAC1**. Total proteins from wild-type strain (lane a) and TrLAC1 transformant (lane b) were loaded onto an SDS-PAGE (11% polyacrylamide) gel. Gel was stained with Coomassie blue. L, molecular mass standards.

The electrophoretic mobility of the total secreted proteins in the culture medium was checked on SDS-PAGE (Figure 1). Compared against the negative control (supernatant from wild-type strain, Figure 1, lane a), an additional band was visualized at ~104 kDa in the TrLAC1 transformant (Figure 1, lane b). Recombinant TrLAC1 was purified through a one-step chromatographic strategy (IMAC). The homogeneity of the recombinant TrLAC1 was controlled by SDS-PAGE (Figure 1, lane c) and a single ~104 kDa band was detected. The deduced molecular mass of the predicted mature TrLAC1 is ~67 kDa, thus the experimental molecular mass of TrLAC1 could originate from variable mobility in SDS gel analysis and cellular processing such as glycosylations (the mature sequence reveals 6 consensus sites for *N*-glycosylation). Identification of the recombinant TrLAC1 was confirmed by tryptic digestion followed by MALDI-TOF analysis (data not shown). When the extracellular protein patterns of the parental strain and the best tranformants (Figure 1, lanes a and b) were compared, it is evident that the most abundant band corresponding to the cellobiohydrolase CBHI is missing from the transformant. This indicates that the expression construct has replaced the *cbh1 *gene by homologous recombination in this transformant.

#### pH and temperature optimum of TrLAC1

**Figure 2:**
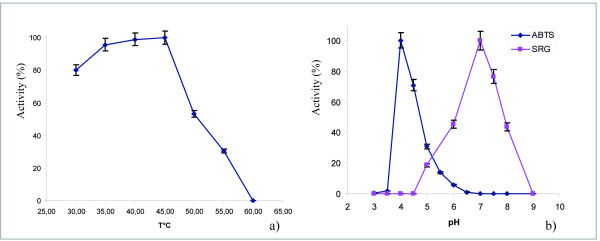
**Effect of temperature (a) and pH (b) on the activity of the purified TrLAC1**. Various temperatures and pH values were tested under standard conditions by using ABTS "blue diamond" and SRG "pink square" as substrates.

The optimum temperature and pH of TrLAC1 was measured by assaying its activity at different temperature and pH values using ABTS as a substrate. The optimum temperature was 40-45°C and optimum pH was 4 (Figure 2a and 2b). Activity towards the canonical laccase substrate syringaldazine (a phenolic compound) was also determined and optimum pH was pH 7.

#### Effect of pH value and temperature on the residual activity of TrLAC1 after incubation

**Figure 3:**
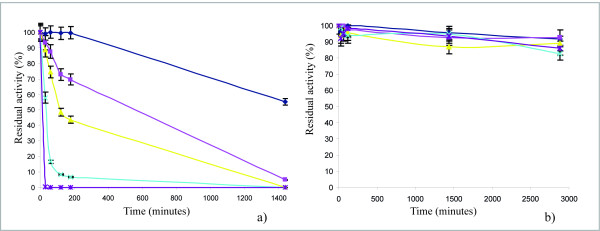
**Effect of temperature (a) and pH (b) on the stability or the purified TrLAC1**. Selected temperatures were 30°C "blue diamond", 35°C "pink square", 40°C "yellow triangle", 45°C "blue cross", 50°C "purple star" and the selected pH values were 3 "blue diamond", 4 "pink square", 5 "yellow triangle", 6 "blue cross", 7 "purple star".

TrLAC1 was incubated for different times at different temperature and pH values and subsequently the residual activity was measured. The enzyme conserved its activity at 30°C after 3-hr incubation but TrLAC1 lost approximately half of its activity after 24 h (Figure 3a). TrLAC1 is not a thermostable enzyme. At 35°C and 40°C, 30% and 66% of enzymatic activity, respectively, was lost. At 45°C, laccase activity was totally lost after 2 hours. However, TrLAC1 showed good stability within a pH range of 3 to 7 (Figure 3b).

#### Kinetic properties

The kinetic parameters of the recombinant TrLAC1 were determined using Lineweaver-Burk plot method by using ABTS as substrate (Table 1). *K*_m_, and *V*_max _were determined from Lineweaver-Burk plot analysis as 0.137 mM and 9.37 nkat.mL^-1^, respectively. Specific activity equals 2.13 nkat.mg^-1^.

**Table 1:** Properties of TrLAC1 from *T. reesei*

**Molecular weight (KDa)**	104
**pH optimum**	4
**Tp optimum (°C)**	40-45
***K***_***m ***_**(mM)**	0.137
***V***_***max ***_**(nkat/ml)**	9.37
**Specific activity (nkat/mg)**	2.13

#### Circular dichroism

**Figure 4:**
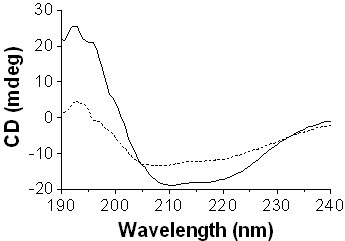
**CD spectra of TrLAC1**. measured under native (solid line) and thermal denaturing conditions (dashed line).

The far-UV CD spectra of TrLAC1 under native and thermally denaturing conditions were compared as an indicator of the secondary structure. Figure 4 showed that the spectra measured at 25°C differed from the spectra measured at 90°C, suggesting that the native TrLAC1 exist in a conformational state with significant secondary structure. Moreover, thermal stability (30 to 90°C) was assessed using CD by following the change at 222 nm (data not shown). Although very broad with no clear folded-unfolded transition state, it was evident that the protein is not thermostable, which is in agreement with the data from the residual activity measurements.

### Evolutionary analyses

#### Phylogenetic analyses

**Figure 5:**
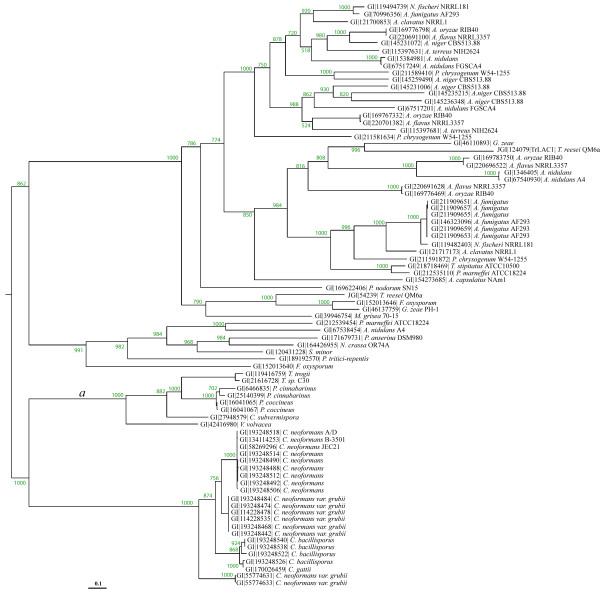
**Phylogenetic reconstruction of homologous sequences for TrLAC1**. Basidiomycotina laccases branch is labelled as *a *and is considered as the foreground branch for branch-site models.

All the homologous proteins of TrLAC1 from *T. reesei *were selected from non-redundant databases and integrated into phylogenetic analysis using maximum likelihood for phylogenetic reconstruction. The high bootstrap values confirmed robust tree topology. Phylogenetic analysis clearly differentiated two distinct groups containing the laccases of Basidiomycota and Ascomycota, TrLAC1 belonging to the latter group. According to the tree topology, Basidiomycota and Ascomycota groups belong to two different evolutionary histories leading to high sequence divergence between homologs. The Ascomycotina group is split into two subgroups with a high bootstrap value of 862 (Figure 5). No complete functional characterization has yet been carried out in the Ascomycotina group. This study on TrLAC1 provides therefore a biochemical investigation in the Ascomycotina group that could facilitate future functional annotation in the clade by functional inference.

The Basidiomycotina group is divided in two subgroups (bootstrap values of 1000) including the classical laccases (*Pycnoporus*, *Trametes*) involved in ligninolysis and the laccase from the pathogenic yeast C*ryptococcus neoformans *involved in the biosynthesis of melanin as a virulence factor.

#### Evolutionary analyses

In order to shed light on the differences between classical laccases and LMCO from Ascomycotina as studied in this work, we hypothesized that positive selection occurred along the lineage leading to classical Basidiomycotina laccases, and the branch was labelled *a*. The purpose of this analysis was to test for positive selection along the branch leading to ligninolytic enzymes, i.e. laccase, and to evidence sites under positive selection.

The branch-site model A was applied to test evolutionary shift by maximum likelihood analysis. Branch *a *was considered as the foreground branch and all others as background branches in the phylogenetic tree. Parameter estimates under model A suggested that 39.3% of sites were under positive selection with ω_2 _= 14.2, whereas 10.2% of sites were under the neutrality assumption with ω_1 _= 1 fixed, and finally 50.3% of sites were highly conserved with ω_0 _= 0 (Table 2). The likelihood ratio tests (LRTs) gave 2*Δl *= 33.78 with P < 10^-4 ^and df = 2 for the test comparing model A to model M1a (neutral) and 2*Δl *= 6.84 with P < 10^-2 ^and df = 1 for the test comparing model A to model A with ω_2 _= 1 fixed. Therefore, LRTs confirmed that results were highly significant.

**Table 2:** Parameter estimates for the tree

Model	*p*	*l*	Parameter estimates	Positively-selected sites
M0: one-ratio	1	-5974.07	ω = 0.0621	None
Branch-site model:				
Model A	3	-5925.68	*p*_0 _= 0.503, *p*_1 _= 0.102	Site for foreground lineage:
			(p_2 _+ *p*_3 _) = 0.393	100P 102F 115L 131S 137Y 138C 158Y 223S 279W 339E 349L 368P
			ω_2 _= **14.2**	379G 388P 393F 455N
				(at *P *> 0.9)

Note: *p *is the number of free parameters for the ω ratios. Parameters indicating positive selection are presented in boldtype. Sites potentially under positive selection were identified using laccase from *P. cinnabarinus *(AAF13052) according to Bayes Empirical Bayes analysis of Model A.

These results demonstrated that positive selection occurred, and the Bayes empirical Bayes (BEB) test identified 16 sites under positive selection along the branch leading to classical laccases, at a probability level of >90%. These results show that positive selection drove the evolution of sequences leading to well-known laccases involved in ligninolysis. Among these sites, 14 are not conserved in the TrLAC1 sequence, meaning that these sites could be targeted for mutagenesis and functional studies between classical laccases and LMCO from *T. reesei *(and more generally from Ascomycotina).

## Discussion

A large number of microbial genomes relevant to lignocellulose degradation are currently being sequenced [36]. Among them, *T. reesei *is a model organism of fungal decay of cellulose and hemicellulose, and its enzymes were demonstrated to have potential in production of bioethanol and other biological products from biomass. *T. reesei *is widely used for its unique secretion ability, especially for cellulase production. *T. reesei *fungus possesses 10 genes encoding well-known cellulases (endoglucanases and cellobiohydrolases) and 16 genes that encode hemicellulase genes classified in several CAZyme families [3,37]. *T. reesei *has never before been considered as a potentially lignin modifying fungus. The study of an endogenous LMCO in *T. reesei*, the biotechnological workhorse of the genus *Trichoderma*, is therefore of interest for the multiple biotechnological applications using this fungus and is important in an ecological context [38].

In literature, there are several records of laccases production by ascomycetes. In the genus *Trichoderma *(*T. atroviride *and *T. harzianum*), laccase activity was correlated with the production of the green pigment in conidial spores [39]. Laccase activity has also been evidenced in the tropical xylariaceous fungi that are probably responsible for the lignocellulolysis despite their slow decay ability *in vitro *[40]. In addition, laccases were purified from phytopathogenic ascomycetes such as *Gaeumannomyces graminis *[41], *Magnaporthe grisea *[42]*Ophiostoma novo-ulmi *[43]*Mauginiella *[44]*Monocillium indicum *[45], *Neurospora crassa *[46].

Concerning recombinant enzyme production, the expression of an ascomycete laccase-encoding gene from *Melanocarpus albomyces *was reported in *T. reesei *and the recombinant protein characterized [21]. Additionally, heterologous expression of a thermostable laccase from the ascomycete *Myceliophtora thermophila *was successfully achieved in *Aspergillus oryzae *[47].

Fungal LMCOs could play physiological roles in lignin degradation in addition to other functions, such as iron metabolism, pathogenic interactions, pigment synthesis in conidiospores, in the induction of fruiting bodies and in offensive/defensive strategies during interactions of fungi [17-20,48,49].

The annotation of *T. reesei *genome using the FOLy database revealed the presence of laccase-like multicopper oxidase genes (LMCO) related to laccase sequences from Basidiomycetes (Levasseur *et al*., 2008a). To date, no expression of these genes has been evidenced in *T. reesei*, and it could be hypothesized that a specific expression pattern strictly constraints expression.

Homologous overproduction assays were conducted for the gene jgi|124079|. The supernatant of the transformed strain showed laccase activity and the corresponding recombinant protein was named TrLAC1. Biochemical characterization revealed that this enzyme is active on typical specific laccase substrates including ABTS and syringaldazine. Interestingly, TrLAC1 has a higher pH optimum spectrum on syringaldazine compared to most of the other basidiomycete laccases (i.e. *Pycnoporus cinnabarinus*). The optimal pH of TrLAC1 is similar to laccases from the basidiomycete *Cyathus stercoreus *and the ascomycete *Melanocarpus albomyces *that have been reported to have optimal pH of 6-7 by using syringaldazine [50,51].

Interestingly, optimum pH differed strikingly according to the substrate used as the optimum pH was 7 with the phenolic substrate syringaldazine and 4 with the nonphenolic substrate ABTS. Such a behaviour has already been reported for other fungal laccases and could be explained by two phenomena: one generated by the redox potential difference between a reducing substrate and the type 1 copper of laccase and another generated by the binding of a hydroxide anion to the type 2/type 3 coppers of laccase [52,53].

In a biotechnological point of view, TrLAC1 represents a candidate for applications at alkaline pHs such as bioremediation of textile wastewaters and of wastewaters produced by chemical plants in the manufacturing organic molecules [54]. Nevertheless, TrLAC1 had a low specific activity on ABTS as compared to basidiomycete laccases. For instance, *P. cinnabarinus *laccase has a specific activity ~800 times higher on ABTS than TrLAC1 [55]. Positively selected sites in TrLAC1 could be specifically targeted in future to study their impact in the enzymatic efficiency.

There are surprisingly few studies focused on the diversity and function of LMCO genes in soil-inhabiting ascomycetes, despite their probable role in plant litter decay processes [56-58]. In order to screen the gene biodiversity of potential laccases from Ascomycotina, a phylogenetic tree was constructed using maximum likelihood analysis. Numerous homologs of TrLAC1 were observed in the Ascomycotina group, especially in the *Aspergilli*. For instance, *Aspergillus niger *CBS513.88 has 5 potential candidate homologous genes similar to LMCO. Interestingly, a paralog was identified in *T. reesei *(jgi|54239|). In an evolutionary point of view, functional characterization of this candidate protein could be useful to understand the evolution of LMCO in *T. reesei *and to unravel the fate of duplicates in genome. Functional characterization has not been performed in the Ascomycotina group, and TrLAC1 characterization could therefore be useful for future evolutionary-based functional annotation [4,59].

In order to shed light on the evolutionary history of laccases, branches leading to well-known ligninolytic laccases were targeted for testing selective pressure. Positive selection was detected, and specific sites were evidenced. Comparing these positively-selected sites to the laccase from Ascomycotina could target potential sites for future directed mutagenesis research. Hybrid laccases combining Ascomycotina sequences and positively-selected sites identified in Basidiomycotina could prove useful for testing new physico-chemical properties for biotechnology applications [60,61]. In addition, testing positively-selected sites could be valuable in drawing a parallel between functional and evolutionary shifts in the multi-copper family.

## Conclusions

*T. reesei*, the biotechnological workhorse of the genus *Trichoderma*, has only been reported as a cellulolytic and hemicellulolytic organism. This work reports homologous overexpression of the first LMCO from *T. reesei *and the biochemical characterization of the recombinant protein. In addition, phylogenetic and evolutionary analyses were carried out to explore the biodiversity of this enzyme group in *Ascomycotina*. Positive selection was detected, and specific sites were evidenced and could be specifically targeted in future to study their impact in the enzymatic efficiency. These results give new insights into the physicochemical parameters and evolution in fungal LMCO.

## Authors' contributions

AL designed and performed the whole analysis, interpreted the results and draw conclusions. MS helped to perform fungal transformation. DN, MA, KK, ER helped to perform biochemical analyses. PP helped to carry out phylogenetic analysis. AL wrote the manuscript. All the authors participated in results analysis, read and approved the final manuscript.
